# Association between occupational or environmental noise exposure and renal function among middle-aged and older Korean adults: a cross-sectional study

**DOI:** 10.1038/s41598-021-03647-4

**Published:** 2021-12-16

**Authors:** You Jin Kim, Won-Jun Choi, Seunghon Ham, Seong-Kyu Kang, Wanhyung Lee

**Affiliations:** 1 College of Medicine, Gachon University, Incheon, Republic of Korea; 2 Department of Occupational and Environmental Medicine, Gil Medical Center, Gachon University College of Medicine, Namdong-daero 774, Incheon, Republic of Korea

**Keywords:** Epidemiology, Chronic kidney disease, Health occupations, Risk factors

## Abstract

Exposure to occupational and environmental noise is closely linked to various auditory system diseases. Few studies have focused on the effect of noise exposure on the extra auditory system, especially the urinary system. We analyzed 17,154 participants aged 40–79 years from the Korea National Health and Nutrition Examination Survey between 2013 and 2018. A self-reported questionnaire was used to assess occupational or environmental noise exposure. Logistic regression was used to determine the differences in the prevalence of chronic kidney disease (CKD) based on noise exposure characteristics. For participants with noise exposure, linear regression was performed to determine relationship of the noise exposure period and estimated glomerular filtration rate (eGFR). In the noise exposure group, a higher CKD prevalence was associated with females who experienced long-term occupational noise (≥ 240 months) (adjusted OR 2.72, 95% CI 1.11–6.66). An increase of one month of occupational noise exposure was associated with a 0.0106 mL/min/1.73 m^2^ decrease in eGFR in females aged < 60 years. Overall, noise exposure may be a risk factor for reduced renal function, especially long-term occupational noise exposure. More precise studies should determine (1) the relationship between noise and renal function and (2) the underlying mechanisms.

## Introduction

Chronic kidney disease (CKD) is a generic term for any condition that leads to kidney damage or decreased renal function. Its severity is assessed using the glomerular filtration rate, albuminuria, and clinical diagnosis^1^. CKD is known to be a risk factor for premature death, cardiovascular disease, stroke, and poor quality of life^2–4^. Globally, the prevalence of CKD is reported to be more than 10%, and its prevalence in Korea is estimated to be 7.9%^5^. The all-cause mortality rate from CKD between 1990 and 2017 increased by 41.5%, ranking as the 12th leading cause of death in 2017^6^. Because of population aging and an increase in diabetes and hypertension (i.e., two of the main causes of CKD), the burden of CKD has intensified^7^. To reduce this burden, determining and avoiding risk factors would be beneficial.

Noise is considered an important risk factor for several diseases^8^. Exposure to noise not only affects auditory acuity, but also leads to non-auditory adverse health outcomes, such as cardiovascular disease, metabolic outcomes, and cognitive impairment^9–11^, especially when there is long-term exposure^8^. The biological mechanism of the non-auditory effects of noise can be explained by the induction of the sympathetic-adrenal-medullary (SAM) axis and the hypothalamic-pituitary-adrenal (HPA) axis. Activation of these axes releases epinephrine, norepinephrine, and cortisol, which alter blood flow and metabolism^12,13^. Environmental noise is defined as the noise created from all sources, except workplaces. Noise exposure in the workplace is called occupational noise^8^.

Despite its known effect, there is a lack of studies on the impact of noise on renal function. Because renal hemodynamics are closely related to blood pressure, vascular reactivity, and endothelial function, renal function might be influenced by noise-induced stress^14^. One study showed that residential proximity to major roadways is related to the reduction of renal function in hospitalized patients with acute ischemic stroke^15^. In addition, a reduction in renal function has been associated with community noise exposure in male patients with cardiovascular disease^16^. However, few epidemiological studies have considered how different types of noise exposure are related to renal diseases.

Therefore, we conducted a cross-sectional study to determine whether noise exposure is associated with renal function. Specifically, we examined the association between the two types of noise exposure status, which are occupational and environmental noise, and renal function (CKD and the estimated glomerular filtration rate [eGFR]). We hypothesized that noise exposure inversely affects renal function.

## Results

### Participants’ characteristics

The participants’ characteristics according to the eGFR are summarized in Table 1. There were 633 (3.7%) participants with CKD (eGFR < 60 mL/min/1.73 m^2^) and the mean eGFR value was 89.8 ± 14.5 mL/min/1.73 m^2^. The older group, aged from 60 to 79 years, had a higher prevalence of CKD (7.5%) than the younger group, aged 40 to 59 years (0.7%). The CKD prevalence in males was 4.7%, which was higher than that in females (3.0%). Regarding educational status, participants who went to college or achieved higher education had the lowest CKD prevalence (1.5%), while those with an elementary school education had the highest prevalence (7.1%). Participants with a higher household income showed a lower CKD prevalence: 1st quartile (8.3%), 2nd quartile (3.9%), 3rd quartile (2.2%), and 4th quartile (1.6%). The CKD prevalence was higher in rural areas (4.8%) than in urban areas (3.4%). Compared to the other groups, the following health conditions were more prevalent in the CKD group: hypertension (HTN) (7.4%), diabetes mellitus (DM) (9.7%), dyslipidemia (5.1%), and high body mass index (BMI) (4.9%). The CKD prevalence according to smoking status, from high to low, was as follows: past smokers (5.1%), never smokers (3.3%), and current smokers (3.2%). The prevalence of CKD was lower in the high-risk alcohol consumption group (1.1%) than in the non-high-risk group (4.0%), and more participants who reported no aerobic physical activity had CKD (4.5%) than those who did not (2.6%). Groups classified according to occupational noise and environmental noise did not show a statistical difference in CKD prevalence or the mean eGFR (*p* > 0.05). Participants who reported hearing discomfort had a higher CKD prevalence (7.3%) than those who did not (3.0%).

**Table 1:** Characteristics of study participants according to the kidney function status.

Characteristics	Total	CKD, *n* (%)		*p*-value^†^	eGFR	*p*-value^‡^
		No	Yes		Mean ± SD	
**Total participants**	17,154	16,521 (96.3)	633 (3.7)		89.8 ± 14.5	
**Socioeconomic status**						
Age (years)				< 0.0001		< 0.0001
40–59	9607 (56.0)	9542 (99.3)	65 (0.7)		96.1 ± 12.0	
60–79	7547 (44.0)	6979 (92.5)	568 (7.5)		81.9 ± 13.5	
Sex				< 0.0001		< 0.0001
Male	7421 (43.3)	7075 (95.3)	346 (4.7)		86.9 ± 14.4	
Female	9733 (56.7)	9446 (97.0)	287 (3.0)		92.1 ± 14.2	
Educational status				< 0.0001		< 0.0001^§^
Elementary school	4478 (26.1)	4161 (92.9)	317 (7.1)		83.6 ± 14.4	
Middle school	2405 (14.0)	2316 (96.3)	89 (3.7)		88.6 ± 13.7	
High School	5459 (31.8)	5305 (97.2)	154 (2.8)		92.5 ± 14.3	
College or higher	4812 (28.1)	4739 (98.5)	73 (1.5)		93.2 ± 13.4	
Household income				< 0.0001		< 0.0001^§^
1st Quartile	3451 (20.1)	3165 (91.7)	286 (8.3)		83.6 ± 15.7	
2nd Quartile	4302 (25.1)	4135 (96.1)	167 (3.9)		89.4 ± 14.3	
3rd Quartile	4465 (26.0)	4366 (97.8)	99 (2.2)		92.3 ± 13.7	
4th Quartile	4936 (26.8)	4855 (98.4)	81 (1.6)		92.4 ± 13.2	
Urbanity				< 0.0001		< 0.0001
Urban area	13,702 (79.9)	13,235 (96.6)	467 (3.4)		90.2 ± 14.4	
Rural area	3452 (20.1)	3286 (95.2)	166 (4.8)		88.6 ± 14.9	
**Chronic diseases**						
Hypertension				< 0.0001		< 0.0001
No	10,439 (60.9)	10,303 (98.7)	136 (1.3)		93.0 ± 12.9	
Yes	6715 (39.1)	6218 (92.6)	497 (7.4)		85.0 ± 15.5	
Diabetes				< 0.0001		< 0.0001
No	14,470 (84.4)	14,096 (97.4)	374 (2.6)		90.8 ± 13.8	
Yes	2684 (15.6)	2425 (90.4)	259 (9.7)		84.6 ± 17.1	
Dyslipidemia				< 0.0001		< 0.0001
No	8332 (48.6)	8148 (97.8)	184 (2.2)		92.3 ± 13.6	
Yes	8822 (51.4)	8373 (94.9)	449 (5.1)		87.5 ± 15.0	
BMI				< 0.0001		< 0.0001
< 25 kg/m^2^	10,905 (63.6)	10,579 (97.0)	326 (3.0)		90.9 ± 14.1	
≥ 25 kg/m^2^	6249 (36.4)	5942 (95.1)	307 (4.9)		88.0 ± 15.2	
**Health behaviors**						
Smoking				< 0.0001		< 0.0001^§^
Never	10,282 (59.9)	9945 (96.7)	337 (3.3)		91.1 ± 14.3	
Past	4008 (23.4)	3804 (94.9)	204 (5.1)		85.6 ± 14.4	
Current	2864 (16.7)	2772 (96.8)	92 (3.2)		91.3 ± 14.5	
Alcohol consumption				< 0.0001		< 0.0001
None or social	15,355 (89.5)	14,741 (96.0)	614 (4.0)		89.4 ± 14.7	
High-risk	1799 (10.5)	1780 (98.9)	19 (1.1)		93.5 ± 12.6	
Physical activity				< 0.0001		< 0.0001
Regular	7355 (42.9)	7162 (97.4)	193 (2.6)		90.6 ± 13.9	
None	9799 (57.1)	9359 (95.5)	440 (4.5)		89.3 ± 15.0	
**Noise exposure**						
Occupational noise				0.1162		0.3066
No	14,239 (83.0)	13,699 (96.2)	540 (3.8)		89.8 ± 14.6	
Yes	2915 (17.0)	2822 (96.8)	93 (3.2)		90.1 ± 14.2	
Environmental noise				0.7165		0.4651
No	16,851 (98.2)	16,228 (96.3)	623 (3.7)		89.8 ± 14.5	
Yes	303 (1.7)	293 (96.7)	10 (3.3)		90.4 ± 13.6	
Hearing discomfort				< 0.0001		< 0.0001
Comfort	14,209 (82.8)	13,790 (97.0)	419 (3.0)		91.0 ± 14.2	
Discomfort	2945 (17.2)	2731 (92.7)	214 (7.3)		84.4 ± 15.1	

*CKD* chronic kidney disease, *SD* standard deviation, *eGFR* glomerular filtration rate, *BMI* body mass index.

^†^Chi-squared test; ^‡^Student’s *t*-test; ^§^One-way analysis of variance.

### Noise exposure and CKD

To determine the association between noise exposure and CKD, logistic regression analysis was performed (Table 2). There was no significant difference in CKD between the noise-exposure and non-exposure groups in the crude and adjusted models. Among those in the noise exposure group, we analyzed effect of long-term noise exposure, which was set as the upper 25%. There was no association between long-term environmental noise exposure and CKD; however, for occupational noise, there were statistical associations in the crude model for the total group (OR 2.34, 95% confidence interval [CI] 1.54–3.54), male group (OR 1.77, 95% CI 1.08–2.89), and female group (OR 3.52, 95% CI 1.54–8.03). The adjusted model only showed significant results in the female group (OR 2.72, 95% CI 1.11–6.66). As noise exposure results in a decrease in hearing ability, we regarded hearing discomfort as an index of crude noise exposure. In the unadjusted model, CKD was significantly associated with hearing discomfort (OR 2.58, 95% CI 2.18–3.06); this tendency was maintained after sex stratification. Females showed a stronger association between hearing discomfort and CKD (OR 3.10, 95% CI 2.42–3.96) than males (OR 2.15, 95% CI 1.70–2.72). After adjustment, the total group (OR 1.25, 95% CI 1.04–1.51) and female group (OR 1.42, 95% CI 1.09–1.85) still showed an association. However, there was no significant difference between hearing discomfort and CKD in male (*p* = 0.4474).

**Table 2:** Results of the logistic regression analysis for noise exposure and CKD.

	Crude			Adjusted		
	OR	95% CI	*p*-value	OR	95% CI	*p*-value
**Occupational noise**						
Total	0.84	0.67–1.05	0.1168	0.91	0.72–1.16	0.4592
Male	0.79	0.60–1.03	0.0859	0.99	0.73–1.33	0.9346
Female	0.71	0.47–1.06	0.0933	0.78	0.51–1.19	0.2422
**Long-term occupational noise**^**†**^						
Total	2.34	1.54–3.54	< 0.0001	1.55	0.97–2.47	0.0684
Male	1.77	1.08–2.89	0.0235	1.33	0.77–2.29	0.3088
Female	3.52	1.54–8.03	0.0028	2.72	1.11–6.66	0.0287
**Environmental noise**						
Total	0.89	0.47–1.68	0.7167	0.85	0.43–1.67	0.6358
Male	0.98	0.43–2.24	0.9643	0.97	0.40–2.39	0.9544
Female	0.78	0.29–2.12	0.6272	0.73	0.26–2.06	0.5543
**Long-term environmental noise**^**‡**^						
Total	1.46	0.40–5.29	0.5675	0.93	0.17–4.99	0.9284
Male	1.34	0.23–7.64	0.7430	< 0.001	< 0.001–34.40	0.1462
Female	1.90	0.26–13.8	0.5275	3.38	0.31–37.39	0.3205
**Hearing discomfort**						
Total	2.58	2.18–3.06	< 0.0001	1.25	1.04–1.51	0.0154
Male	2.15	1.70–2.72	< 0.0001	1.11	0.85–1.43	0.4474
Female	3.10	2.42–3.96	0.0001	1.42	1.09–1.85	0.0090

Adjusted for age, sex, educational state, household income, urbanity, hypertension, diabetes, dyslipidemia, body mass index, smoking, high-risk alcohol consumption, and aerobic physical activity.

The cut-off value of long-term exposure is the third quartile of the noise exposure period.

*OR* odds ratio, *CI* confidence interval, *CKD* chronic kidney disease.

^†^Only for participants with occupational noise exposure (*n* = 2915). “Long-term” refers to occupational noise exposure for ≥ 240 months.

^‡^Only for participants with environmental noise exposure (*n* = 303). “Long-term” refers to environmental noise exposure for ≥ 300 min per day.

### Noise exposure and eGFR

The linear analysis results for the noise exposure time and eGFR are presented in Table 3. We only analyzed the noise exposure group; thus, there were 2915 participants in total (1720 males and 1195 females) in the occupational noise group. In the environmental noise group, there were 303 participants (131 males and 172 females). For occupational noise, only the crude models showed an inverse association between noise exposure time and eGFR. We then stratified patients according to age: < 60 years and > 60 years. In the < 60 year age group, there were inverse associations of noise exposure and eGFR with the crude models. After adjustment, a one-month increase in occupational noise in females was associated with 0.0106 (± 0.0052) mL/min/1.73 m^2^ decrease of eGFR. For patients aged ≥ 60 years, the crude model of the total group showed an inverse association (− 0.0056 [± 0.0026] mL/min/1.73 m^2^) between occupational noise period and eGFR, but no significant results were observed after stratification by sex and adjustment. In the environmental noise group, there were no significant results for any model.

**Table 3:** Results of the linear regression analysis for noise exposure time and the eGFR.

	Crude			Adjusted		
	B	SE	*p*-value	B	SE	*p*-value
**Occupational noise**						
Total (*n* = 2915)	− 0.0199	0.0019	< 0.0001	− 0.0020	0.0017	0.2551
Male (*n* = 1720)	− 0.0135	0.0023	< 0.0001	− 0.0013	0.0021	0.5234
Female (*n* = 1195)	− 0.0276	0.0040	< 0.0001	− 0.0053	0.0034	0.1159
**Environmental noise**						
Total (*n* = 303)	− 0.0022	0.0041	0.5923	− 0.0013	0.0036	0.7205
Male (*n* = 131)	− 0.0030	0.0063	0.6292	0.0030	0.0057	0.6025
Female (*n* = 172)	− 0.0028	0.0054	0.6050	− 0.0028	0.0048	0.5552
**40 ≤ age < 60**						
Occupational noise						
Total (*n* = 1699)	− 0.0142	0.0025	< 0.0001	− 0.0019	0.0025	0.4562
Male (*n* = 1015)	− 0.0062	0.0030	0.0392	− 0.0008	0.0029	0.7771
Female (*n* = 684)	− 0.0185	0.0054	0.0006	− 0.0106	0.0052	0.0416
Environmental noise						
Total (*n* = 171)	0.0033	0.0049	0.5014	0.0029	0.0047	0.5374
Male (*n* = 73)	0.0035	0.0072	0.6297	0.0073	0.0072	0.3118
Female (*n* = 98)	0.0017	0.0066	0.7952	0.0008	0.0063	0.9049
**60 ≤ age < 80**						
Occupational noise						
Total (*n* = 1216)	− 0.0056	0.0026	0.0317	− 0.0025	0.0025	0.3092
Male (*n* = 705)	− 0.0012	0.0032	0.7043	− 0.0020	0.0029	0.4827
Female (*n* = 511)	− 0.0031	0.0051	0.5466	− 0.0022	0.0048	0.6520
Environmental noise						
Total (*n* = 132)	− 0.0050	0.0057	0.3815	− 0.0043	0.0057	0.4508
Male (*n* = 58)	− 0.0069	0.0090	0.4457	0.0082	0.0096	0.3985
Female (*n* = 74)	− 0.0045	0.0071	0.5315	− 0.0084	0.0081	0.3062

Adjusted for age, sex, educational state, household income, urbanity, hypertension, diabetes, dyslipidemia, body mass index, smoking, high-risk alcohol consumption, and aerobic physical activity.

*eGFR* glomerular filtration rate, *SE* standard error.

## Discussion

This study aimed to determine the association between noise exposure and renal function. Regarding occupational noise, long-term exposure was associated with CKD prevalence in the unadjusted model; however, in the adjusted model, the association was only observed among females (OR 2.72, 95% CI 1.11–6.66; Table 2). Linear regression was used to examine the dose–response relationship between noise exposure and eGFR. An inverse relationship between occupational noise exposure time and eGFR was found in females under 60 years of age (− 0.0106 [± 0.0052] mL/min/1.73 m^2^; Table 3). It seems that for old age (60 to 79 years), the effect of age to eGFR might be larger than that of noise exposure. No relationships between environmental noise exposure and renal function were found in the logistic or linear analyses. We found that hearing discomfort was related to CKD prevalence (OR 1.25, 95% CI 1.04–1.51; Table 2). That is, participants who complained of hearing discomfort had a higher CKD rate. When stratified by sex, this tendency persisted among female participants (OR 1.42, 95% CI 1.09–1.85; Table 2).

There have been few studies on the relationship between noise and renal function. An epidemiological study of 1103 patients from the Boston area in the United States who had ischemic stroke showed lower eGFR as they live closer to a major roadway^15^. Moreover, in 217 patients with cardiovascular heart disease, increased exposure to day–evening–night noise levels (L_den_) resulted in a decrease in eGFR among men who experienced ischemic heart disease or stroke and who were exposed to lower air pollution^16^. Our findings are similar to those of these studies in terms of noise exposure. However, our results do not overlap with those of previous studies from the perspective of environmental noise. Previous studies have focused on environmental noise and the lack of occupational exposure to noise. In our study, we analyzed both environmental and occupational noise and found no significant relationship between renal function in environmental noise but occupational noise. This inconsistency might be caused by the low number of respondents with noise exposure.

Other studies produced results opposite to those in our study. A randomized single blinded control study compared renal hemodynamics after aircraft noise and sham procedures and found no significant changes in renal circulation, including the GFR^17^. In addition, a cross-sectional single-center study found an association between noise annoyance, renal perfusion, and renal vascular resistance; however, there was no difference in measured GFR by noise annoyance^18^. Because the study populations and noise characteristics differed from those in our study, the results also differed. For both studies, the study participants were all men who were approximately 30–50 years of age; our data, however, included participants of both sexes who were 40–79 years old.

Until now, the non-auditory effects of noise have been investigated in terms of cardiovascular, metabolic, and cognitive outcomes^9–11^. The probable mechanisms have been suggested from many perspectives. First, noise acts as a stressor that induces sympathetic nerve activity. The stress responses are mediated by the HPA and SAM axes. The SAM axis secretes catecholamines as a reaction to acute stress, while the HPA secretes glucocorticoids, such as cortisol, which prolongs stress^19^. These can alter glucose metabolism, increase blood pressure and free fatty acids, inhibit insulin, and adversely affect lymphocytes^20,21^. In addition, inflammation and oxidative stress can be activated by both axes, leading to endothelial dysfunction^19^. As renal function is influenced by vascular function^22^, the non-auditory effects of noise exposure on renal function may share similar mechanisms. Recently, a possible explanation has been provided from the perspective of epigenetic transformation. In epigenome-wide association studies using SAPALDIA data, some CpG sites related to C-reactive protein (CRP), BMI, and eGFR were methylated in the noise exposure group^23^. These explanations can help to elucidate the relationship between renal function and noise exposure.

The female group, especially in the middle-aged group (40–59 years), seemed to show a stronger association between noise exposure and renal function. The results of our logistic regression on hearing discomfort and long-term occupational noise exposure showed an OR > 1 in the female group (Table 2). In addition, the linear regression coefficients were significant, but only in females under 60 years of age (Table 3). Considering the sexes, we can assume that females are more vulnerable to noise-induced stress in terms of biological mechanisms. This is because our results are in line with the general concept that there are more stress-related psychiatric disorders among women^24,25^. In addition, according to an analysis in European countries, there was a significant elevation of salivary cortisol levels among females in relation to aircraft noise, but not among males^26^. However, there have been conflicting results. Several studies have found that noise exposure has a more severe impact on males^27,28^. As the results are different by types of stress, population characteristics, biomarkers, and many other elements^29–31^, more research is needed to clarify the interpretation.

Despite its significant contributions, this study has several limitations. First, there were limitations due to the cross-sectional design. Specifically, we were unable to confirm causal relationships, and the order of events could not be considered; logically, we believe that noise exposure influences the reduction in renal function, but not vice versa. Additional studies are required to clarify this relationship. Second, although we used a national representative dataset from a survey conducted and managed by the Korea Disease Control and Prevention Agency, the results were not free from bias, such as recall and nonresponse bias. In particular, the noise exposure survey was self-reported; thus, the responses may have been under- or over-exaggerated depending on one’s personality. In the case of environmental noise, participants might not be aware of it because people hear noise in daily life and are used to it; thus, the number of respondents would be reduced. Third, there may be a misclassification by a matter of definition. For example, the eGFR was calculated using a single measurement of serum creatinine levels. As the precise definition of CKD involves abnormalities of kidney structure or function for at least three months^1^, misclassification can occur. However, this is the first method used in clinical practice to classify CKD. Finally, there are no direct measurements or factors that can estimate noise exposure. Since the Korea National Health and Nutrition Examination Survey (KNHANES) uses a self-report questionnaire to evaluate noise exposure, it does not reflect various characteristics of noise, such as loudness, frequency, and duration. In addition, sources of noise such as transportation or outdoor activity could not be assessed. Further studies using real noise data or modeling ambient noise based on residential addresses are required.

Overall, noise exposure was found to be associated with decreased renal function (especially in females) according to our logistic and linear regressions of Korean middle-aged and older populations. The long-term occupational noise exposure group in females showed higher CKD prevalence, and females aged 40–59 years showed decreased eGFR as they exposed to longer period. The results of our study suggest that noise exposure might be a risk factor for reduced renal function. Future studies should aim to determine the relationship between noise and renal function and examine the underlying mechanisms.

## Methods

### Study participants

The KNHANES data from 2013 to 2018 were used in this study. The KNHANES is a nationwide, cross-sectional survey that is conducted in the Republic of Korea to assess the health and nutritional status of Koreans. Representatives were selected using multistage cluster sampling. Annually, 20 households throughout 192 regions are included as a new sample, and approximately 10,000 individuals aged one year and older are targeted. This survey provides participants’ information based on a health examination, health interview, and nutrition survey conducted by trained staff members^32^.

**Figure 1:**
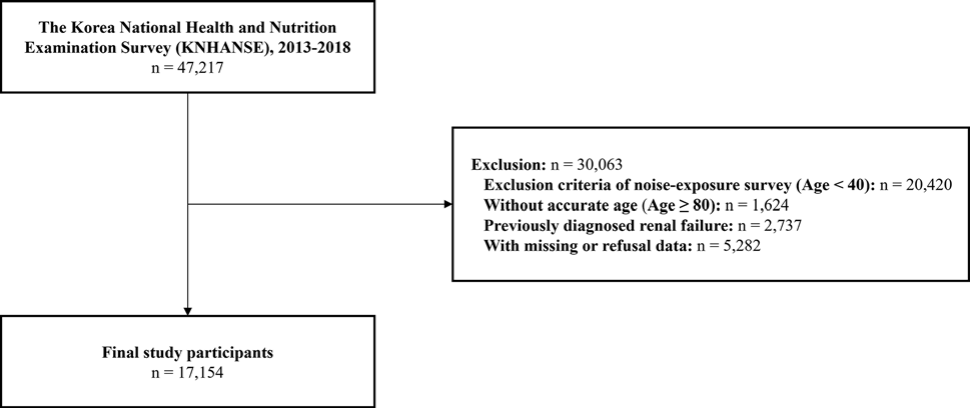
Flow chart of the study participants.

The total number of KNHANES participants from 2013 to 2018 was 47,217. We only included participants aged 40 to 79 years old, as the noise exposure survey was performed at 40 years of age or older and the survey recorded all patients aged 80 years or older as 80 years old. Participants with a previous diagnosis of renal failure were excluded to minimize the effects of kidney disease intervention. Missing or “participant refusal” values of the variables used in this study—such as hearing discomfort status, occupational noise exposure, environmental noise exposure, serum creatinine, educational level, household income, urbanity, HTN, DM, dyslipidemia, BMI, smoking status, high-risk alcohol consumption, and aerobic physical activity—were also excluded. The final sample size for the analysis was 17,202 (Fig. 1).

### Renal function

Renal function was evaluated using the serum creatinine level. This was measured with a Hitachi Automatic Analyzer 7600-210 (Hitachi/JAPAN) and CREA reagent (Roche/Germany) using the Jaffe rate-blanked and compensated method. eGFR was calculated using the Chronic Kidney Disease Epidemiology Collaboration equation^33^. An eGFR lower than 60 mL/min/1.73 m^2^ was classified as CKD.

### Noise exposure

The KNHANES survey used self-reported questionnaires to assess noise exposure. For occupational noise, the question was: “Have you ever worked in place with loud noise such as machines or generators for more than three months? A loud noise means that you have to raise your voices for a conversation.” Those who answered yes were asked about their total working period in months. The question addressing environmental noise was: “Have you ever been exposed to loud noise for more than 5 h a week, except for occupational noise? A loud noise means that you have to raise your voices for a conversation, such as cars, trucks, motorcycles, machines, or loud music (ex. singing room, concert hall.” If they answered “yes,” participants were asked for the average amount of exposure per day (in minutes). Long-term exposure to noise was defined as the third quartile of the noise exposure period (≥ 240 months for occupational noise and ≥ 300 min for environmental noise). As noise exposure is closely linked to auditory problems, we conducted an additional analysis of the association between hearing discomfort and CKD or eGFR. For hearing discomfort, the following question was asked: “Among the following, choose the most appropriate sentence to describe your hearing ability (without wearing a hearing aid).” The options were “comfortable,” “a little uncomfortable,” “very uncomfortable,” and “cannot hear at all.” We excluded participants who responded “cannot hear at all” and combined “a little uncomfortable” and “very uncomfortable” as just “uncomfortable.”

### Other covariates

The socioeconomic variables were age, sex, educational status, household income, and urbanity. Education level was classified as elementary school, middle school, high school, college, or higher as the highest level of completed education. Household income was divided into quarters. Since Korea uses different district names based on urbanization, urbanity was classified according to the district.

For chronic disease variables, we included HTN, DM, dyslipidemia, and BMI. HTN was defined as participants who satisfied at least one of the following criteria: (1) systolic blood pressure ≥ 140 mmHg, (2) diastolic blood pressure ≥ 90 mmHg, or (3) diagnosed with hypertension before or those who used drugs for blood pressure control. Participants with any of the following conditions were considered to have DM: (1) fasting glucose level ≥ 126 mg/dL, (2) diagnosed with DM before, or (3) used oral hypoglycemic medications or insulin injections. Pregnant women were excluded because their gestational diabetes was in a transient state. Dyslipidemia was defined according to the 2018 Korean Dyslipidemia Management Guidelines^34^, and participants who were diagnosed before or who used oral drugs were included. The guidelines describe dyslipidemia as (1) total cholesterol ≥ 240 mg/dL, (2) triglyceride ≥ 200 mg/dL, (3) low-density lipoprotein (LDL-C) ≥ 160 mg/dL, or (4) high-density lipoprotein (HDL-C) < 40 mg/dL. LDL cholesterol levels were calculated using the Friedewald equation^35^. BMI was grouped into two categories: (1) < 25 kg/m^2^ and (2) ≥ 25 kg/m^2^.

The health behavioral variables included smoking status, high-risk alcohol consumption, and aerobic physical activity. Smoking status was classified according to the current smoking status: never smoker, past smoker, or current smoker. High-risk alcohol consumption was defined as averaging ≥ 7 drinks at a time and drinking at least twice a week for men, and an average of ≥ 5 drinks at a time and drinking at least twice a week for females. Aerobic physical activity refers to performing moderate-intensity physical activity (≥ 2.5 h), high-intensity physical activity (≥ 1.25 h), or mixing moderate- and high-intensity physical activity (1 min of high-intensity physical activity is equivalent to 2 min of moderate-intensity) per week.

### Statistical analysis

We conducted a χ^2^ test to examine the differences in CKD prevalence. A Student’s *t*-test and one-way analysis of variance (ANOVA) were used to show the variances in eGFR according to the sociodemographic characteristics. We assessed the association between CKD and noise exposure using logistic regression adjusted for age, sex, educational status, household income, urbanity, HTN, DM, dyslipidemia, BMI, smoking status, high-risk alcohol consumption, and aerobic physical activity. Linear regression analysis was performed to examine the association between eGFR and noise exposure time. Participants who were not exposed to noise were excluded from the analysis. The adjusted covariates were the same as those described above. Statistical analyses were performed using SAS version 9.4 (SAS Institute, Cary, NC, USA). The flowchart as Fig. 1 illustrated by Microsoft 365 Excel.

### Ethics approval and consent to participate

The study was performed in accordance with the ethical standards of the Declaration of Helsinki (1964) and its subsequent amendments. KNHANES data were anonymized prior to their release to the authors. All participants provided written informed consent. The Institutional Review Board of Gil Medical Center, Gachon University, approved this study (IRB number: GCIRB2020-147).

## Data Availability

Data are openly available in a public repository (Korea National Health & Nutrition Examination Survey, https://knhanes.kdca.go.kr/knhanes/eng/index.do).
